# A focus shift in the evaluation of misinformation interventions

**DOI:** 10.37016/mr-2020-124

**Published:** 2023-10-05

**Authors:** Li Qian Tay, Stephan Lewandowsky, Mark J. Hurlstone, Tim Kurz, Ullrich K. H. Ecker

**Affiliations:** (1) School of Psychological Science, University of Western Australia, Australia; (2) School of Psychological Science, University of Bristol, UK; (3) Department of Psychology, Lancaster University, UK; (4) Public Policy Institute, University of Western Australia, Australia

## Abstract

The proliferation of misinformation has prompted significant research efforts, leading to the development of a wide range of interventions. There is, however, insufficient guidance on how to evaluate these interventions. Here, we argue that researchers should consider not just the interventions’ primary effectiveness but also ancillary outcomes and implementation challenges.

## Introduction

A range of recent events has demonstrated the potential for misinformation to incur costly consequences for individuals and societies (e.g., health behaviours) (Bursztyn et al., 2020; Council of Canadian Academies, 2023; Loomba et al., 2021; Simonov et al., 2021). Accordingly, research on interventions to counter misinformation has seen a rapid increase (see Ecker et al., 2022; Kozyreva, Lorenz-Spreen et al., 2022). Whilst these efforts have led to important insights, one critical question remains underexplored: how should misinformation interventions be evaluated? Considering that real-world implementation of interventions inevitably involves trade-offs, holistic assessment is necessary to ensure that agencies and communicators do not waste resources on interventions that may be ineffective or, at worst, backfire. To this end, traditional approaches that (1) apply the common practice of intervention-effect maximization and (2) rely mainly on questionnaire measures to gauge intervention impact are likely insufficient. We, therefore, call for a shift in the focus of assessments of misinformation interventions.

## The need for a focus shift

Misinformation and interventions exist in a complex, multidimensional space (Tay et al., 2023). First, there are different types of misinformation. For example, “bullshit” refers to a type of misinformation with complete disregard for truth, whereas “paltering” refers to misinformation that falls short of literal falsity (Lewandowsky et al., 2017; McCright & Dunlap, 2017). Second, there are different potential outcome variables, such as levels of false belief or polarization (also see Bail et al., 2018). Different types of misinformation may have different causal effects on different outcome variables. For example, bullshit may have lower impact on false beliefs than paltering, but both types may cause polarization. Third, interventions may work for one type of misinformation but not others, and interventions may affect one type of outcome variable but not affect (or have an unintended or delayed effect on) another. For example, interventions that are effective at lowering the impact of both bullshit and paltering on false beliefs may potentially increase polarization (e.g., Democrats being better able to discern misinformation from Republicans could lower their opinions of Republicans and vice versa).

Therefore, when assessing interventions, it is important that researchers and practitioners carefully consider relevant misinformation type(s), outcome variable(s), and causal impacts of both the misinformation and the intervention. Adequate assessment will then need to evaluate not only primary effectiveness but also ancillary impacts and implementation challenges. Whereas existing studies have tended to focus on a limited set of misinformation (e.g., bullshit) and outcomes (e.g., questionnaire measures of misinformation reliance), our proposed perspective can offer a more differentiated framework for intervention evaluation. Below, we first outline and then apply this framework to provide a brief retrospective evaluation of some commonly proposed interventions. However, the framework can also guide the design of novel, proposed interventions.

## How should misinformation interventions be evaluated?

Table 1 provides an overview of our framework. We argue that the complex considerations required for intervention evaluation can be usefully reduced to the aforementioned three dimensions.

Having introduced our framework, we now review some commonly proposed interventions to reveal recent advancements and remaining gaps.

### Post-exposure correction

Post-exposure corrections seek to retroactively reduce misinformation impacts (e.g., fact-checking; debunking). Such interventions tend to reduce the influence of misinformation on beliefs and reasoning, and research has identified several factors that can enhance their effectiveness (see Ecker et al., 2022). Nonetheless, studies in this space typically only focus on explicitly false misinformation and cognitive outcomes, and seldom recruit from the target populations that may be of primary interest to practitioners (for exceptions, see Tay et al., 2022, which included behavioural measures, or Paynter et al., 2019, which embedded debunking of autism myths in a professional-development program for individuals working with pre-school children with autism). Potential impacts on ancillary outcomes often remain unclear; for example, although it generally seems safe to repeat misinformation within a correction in terms of belief impacts (Ecker et al., 2020), corrections may contribute to the amplification of misinformants by using their framing and giving them a platform. There has also been little exploration of implementation challenges, including issues of scalability and real-world efficacy (e.g., best-practice recommendations may be difficult to apply; interventions may not reach individuals most at risk).

### Pre-exposure mitigation

Pre-exposure mitigation aims to pre-emptively reduce individuals’ susceptibility to misinformation. Such interventions include media-literacy treatments (Guess et al., 2020) as well as inoculation—a technique that teaches consumers to recognize flawed reasoning (Lewandowsky & van der Linden et al., 2021). Inoculation research has considered both primary and ancillary outcomes. For instance, recent work has sought to create mobile and web applications that implement inoculation and evaluate its primary effectiveness in public education contexts (e.g., Cook et al., 2022; Roozenbeek & van der Linden, 2019). Studies have also explored so-called post-inoculation talk, a positive ancillary outcome, viz. boosting people’s confidence to speak up about the target issue and thus spreading resistance (e.g., Ivanov et al., 2012). Still, there is need for additional research into potential negative ancillary consequences (e.g., to what extent inoculation enhances scepticism generally rather than increase discernment of true vs. false information; e.g., see Modirrousta-Galian & Higham, 2022, who investigated this using signal detection theory), as well as approaches that can increase uptake of interventions for individuals most vulnerable to misinformation.

### Trust-based interventions

Trust-based interventions aim to foster trust in sources of high-quality information, with a focus on promoting true information rather than debunking false information (e.g., Acerbi et al., 2022). For such proposals, one concern is that sources can be trustworthy on certain topics but spread misinformation on other topics or at other times. For instance, a medical doctor can be trustworthy for the diagnosis of illness but lack the expertise to comment on epidemiological questions, and a news organization that is relatively impartial for domestic affairs can be biased when reporting about other nations. Indeed, many ostensibly trustworthy institutions have engaged in behaviours that warrant scepticism (e.g., mainstream coverage of the alleged “weapons of mass destruction” in Iraq before the invasion of 2003 or climate denialism: Boussalis & Coan, 2016; Calabrese, 2005; scientific misconduct: Stroebe et al., 2012). Nevertheless, one could still argue that increasing trust in mainstream news sources (or science) can generally be expected to yield net benefits, especially in low-trust environments (e.g., it may reduce the likelihood of people consuming information from low-trust sources, where they are relatively more likely to encounter misinformation). However, we caution against generalising such conclusions without first considering the ancillary outcomes that might apply in each context. For example, consider a source that provides 90% true information but, in the remaining 10%, spreads misinformation about a particular minority group. Focusing only on the proportional contribution to true vs. false beliefs as a primary outcome, an intervention that increases trust in the source could be highly effective, particularly if starting from a position of low trust, but could lead to discrimination and violence against the targeted minority. Taken together, this means that overly coarse attempts to increase trust can either be unrealistic in practice or promote uncritical trust that may ironically lead to greater misdirection or other undesirable downstream consequences. Rather, interventions should aim to help individuals acquire the skills to identify topic-specific credible sources or instances of expert consensus. Ideally, trust-boosting interventions should thereby go hand-in-hand with actions to objectively improve the trustworthiness of the relevant institution (e.g., improved journalistic standards or more transparent science) (Munafò et al., 2017; Ward, 2019).

### Context-based interventions

Examples of context-based interventions include accuracy nudges and source labelling. Accuracy nudges highlight the importance of information veracity and aim to reduce misinformation sharing that may occur if individuals are inattentive or not thinking about accuracy (e.g., Pennycook et al., 2020). Source labelling provides transparent cues to help individuals assess information quality (e.g., Kozyreva et al., 2020; Nassetta & Gross, 2020). Such interventions directly alter the context in which (mis)information is processed and thus have the benefit of being scalable on social media. Indeed, they have already been implemented by certain platforms (e.g., Twitter labels and hides messages by officials from some countries deemed to be untrustworthy; Weibo displays location data for user comments). However, their effectiveness may be limited to short-format misinformation such as headlines or tweets. Indeed, misinformation that is technically true (but misleading) or misinformation spread by mainstream sources may evade context-based interventions even if spread on social media. Similarly, source labelling is only effective to the extent that source information is a reliable indicator of credibility, and it risks exacerbating pre-existing biases and polarization (e.g., Republicans distrusting sources that are known to be left-wing; Americans distrusting Chinese sources or vice versa). Further, there is concern that such interventions may be misused (e.g., interventions may disproportionately target enemies of the country that the social-media platform is headquartered in), as there has thus far been little research into guardrails that can ensure fairness and transparency.

### Supply-side interventions

Supply-side interventions aim to reduce the circulation of misinformation by targeting its sources, motivated by the idea that only structural changes can result in sustained improvements. Such approaches can be based on regulation, legislation, or social media policies—recent examples include Singapore’s Protection from Online Falsehoods and Manipulation Act 2019 (which enforces a Correction Direction that requires misinformants to issue public statements acknowledging their spreading of misinformation) or social-media de-platforming (e.g., the routine removal of bot and spam accounts and the suspension of Donald Trump on Twitter). Such interventions may be highly effective in terms of primary outcomes, as misinformation sources can be held accountable or removed. However, there may be normative and practical implications that require further assessment (e.g., concerns about censorship and misappropriation) (see also Kozyreva, Herzog et al., 2022). Other creative supply-side interventions have been proposed, with the hope of better balancing intervention efficacy and risks. One example is “ad guarantees” in a market for truth, which signal the veracity of a claim by placing a resource at risk (e.g., a politician offering a reward for anyone disproving, via an adjudicated process, a claim they made) (Van Alstyne, 2022). Still, both effectiveness for specific information types (e.g., paltering may be less amenable to this approach than bullshit) and whether there is sufficient political or business support for the implementation of such policies remain in question.

## Conclusion

In this article, we have proposed an evaluation framework for misinformation interventions. We argue that researchers and practitioners designing and assessing interventions need to more carefully consider the relevant misinformation types, outcome variables, and their causal relations. Based on these considerations, intervention evaluation should then proceed more holistically, taking into account not just primary effectiveness but also ancillary outcomes and implementation challenges. Retrospectively applying this framework to commonly proposed interventions, it is clear that solutions need to be multi-pronged, and more needs to be done to address research-practice gaps. We note, however, that our proposed framework has two key boundary conditions. First, the framework covers only broad, defining questions, whereas guidance on specific steps within the research process was deemed beyond scope (e.g., stimuli selection and data analysis). Second, the framework focuses on applied settings and real-world implications. For those interested in basic research and theory development, such as determining the cognitive processes that underlie each intervention, our framework may hold limited utility. Nevertheless, it is hoped that the current article will provide some guidance for misinformation-intervention design and evaluation, and contribute to more nuanced discussions about misinformation.

## Figures and Tables

**Table 1:** Framework for the evaluation of misinformation interventions.

Dimension	Action	Rationale	Example
Primary effectiveness	- Set specific targets (e.g., target population and intervention goals, including specifics of misinformation type and outcomes).	Misinformation is diverse in types and potential outcomes. Thus, the assumption that any given intervention is effective across all instances is likely untenable.	- Does the intervention (e.g., one that aims to correct high-school students’ false beliefs regarding mental illness) reduce specific relevant misconceptions?
Ancillary outcomes	- Consider normative implications (e.g., can the intervention be fairly implemented? May the intervention disproportionately empower certain groups whilst harming others?). - Consider causal processes across outcomes and time periods (e.g., Can the intervention backfire on ancillary outcomes? Is the intervention likely to have positive effects beyond primary effectiveness?).	Just as misinformation can have impacts beyond specific false beliefs, interventions can also have ancillary impacts. Such impacts can be both negative (e.g., unfairness, polarization, curtailing freedom of speech) and positive (e.g., fostering norms that value truth, enhancing effects of other interventions), and should be taken into consideration. For misinformation that is unlikely to have dire consequences, overly forceful interventions, even if highly effective for primary targets, may potentially do more harm than good (see also Kozyreva, Herzog et al., 2022).	- Evaluate the immediate and lagged effects of the intervention on tangential false beliefs, students’ mental health, social integration, stigma, and academic performance. - Explore the impacts of the intervention across groups (e.g., students from different socioeconomic classes; students with known mental-health issues; minorities; etc.).
Implementation challenges	- Engage stakeholders (e.g., Who are the key decision-makers and intermediaries? What are their concerns?). - Delineate barriers and incentives (e.g., What are the associated costs? What is the base rate of true vs. false information within a given setting? Are the social, political, and economic environments conducive for the proposed intervention?).	Even if an intervention is likely to be effective and unlikely to have undesirable ancillary outcomes, implementation can pose challenges that limit the scale of the impacts. Such challenges can range from physical (e.g., overhead costs) to political (e.g., constraints of the local political climate). Whereas primary research evaluating effectiveness may have the unit of analysis centred on individuals, research evaluating implementation should centre on stakeholders and systems. Likewise, instead of outcomes such as beliefs, outcomes such as cost and rate of adoption should be evaluated (as in clinical implementation studies; Curran et al., 2012).	- Consultations with students, teachers, families, and school administration. - Estimate the prevalence of misinformation on mental illness on social media such as TikTok (e.g., Yeung et al., 2022). - Estimate the costs and scalability across schools.
